# Dissolved Organic Matter in the Global Ocean: A Primer

**DOI:** 10.3390/gels7030128

**Published:** 2021-08-28

**Authors:** Dennis A. Hansell, Mónica V. Orellana

**Affiliations:** 1 Department of Ocean Sciences, RSMAS, University of Miami, Miami, FL 33149, USA; 2 Polar Science Center, Applied Physics Laboratory, University of Washington, Seattle, WA 98105, USA; morellan@uw.edu; 3 Institute for Systems Biology, Seattle, WA 98109, USA

**Keywords:** marine dissolved organic carbon, ocean carbon cycle, marine microgels

## Abstract

Marine dissolved organic matter (DOM) holds ~660 billion metric tons of carbon, making it one of Earth’s major carbon reservoirs that is exchangeable with the atmosphere on annual to millennial time scales. The global ocean scale dynamics of the pool have become better illuminated over the past few decades, and those are very briefly described here. What is still far from understood is the dynamical control on this pool at the molecular level; in the case of this Special Issue, the role of microgels is poorly known. This manuscript provides the global context of a large pool of marine DOM upon which those missing insights can be built.

## 1. Introduction

Dissolved organic carbon (DOC) makes up the second largest bioavailable pools of carbon in the ocean (~660 Pg C (1 Pg = 1 × 10^9^ metric tons); [1]) and is second only to the ~50× larger pool of dissolved inorganic carbon. The size of the reservoir, and its complementary functions as a sink for autotrophically fixed carbon and as a source of substrate to microbial heterotrophs, indicate that DOC plays a central role in the ocean carbon cycle [2]. Identifying the details and mechanisms of that role in the global ocean remains a great challenge; with relevance to this Special Issue, the contribution of marine gels to those mechanisms is essentially unknown. While a critical percentage of DOC (varying from 10% in the coastal ocean [3] to 30% in the Arctic [4]) assemble as microgels (Orellana and Hansell, this issue), their importance in DOC basin-scale dynamics is just beginning to be understood [5]. Marine gels are three-dimensional (3D), colloidal- to micrometer-sized hydrogel networks held together by Ca^2+^ ionic bonds/hydrophobic bonds that assemble spontaneously from marine dissolved biopolymers [3,4,5]; they have been proposed to play a pivotal role in regulating ocean basin-scale biogeochemical dynamics [6].

In this paper, the role of DOC in the ocean carbon cycle is considered in its broadest temporal and spatial scales, largely as a primer for those wishing to understand the global scale dynamics of the pool. The paper begins with an evaluation of the spatial distribution of DOC at the regional and basin scales, in both the surface and deep ocean. In this context, the net production of DOC relative to the distribution and timing of marine primary production is evaluated. It briefly concludes with priorities for present and future research relevant to the role of gels in DOC dynamics. More complete and detailed reviews of DOC dynamics in the ocean are available [7,8,9,10,11,12,13].

## 2. DOC Concentrations and Reactivity

**Figure 1:**
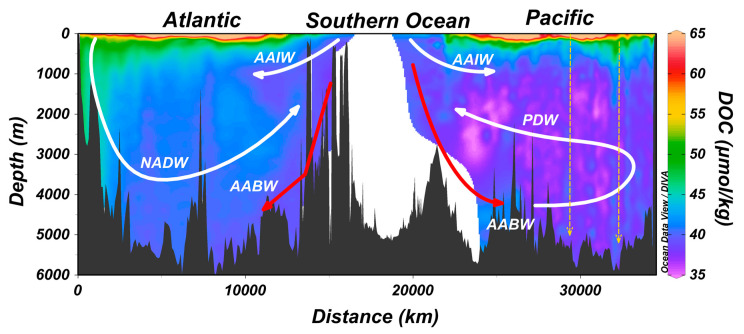
Vertical distribution of DOC (μmolC/kg) along ocean sections A16 and P16 in the Atlantic and Pacific Oceans, respectively (locations given in Figure 2). Data density is low in the Southern Ocean along the section connecting the southern termini of A16 and P16, hence the gap in coverage. Solid arrows schematically indicate the major overturning circulation pathways in the ocean, specifically NADW, AABW, AAIW, and PDW. The dashed arrows represent the apparent pathways of sinking biogenic particles (exported from the surface ocean) that disaggregate while sinking, in turn adding DOC to the deep-water column. This enrichment serves as a substrate to heterotrophs (including prokaryotes) living in the greatest depths of the ocean. Note the vertical alignments of DOC enrichment adjacent to those arrows due to the disaggregation and solubilization of particles. NADW = North Atlantic Deep Water; AABW = Antarctic Bottom Water; AAIW = Antarctic Intermediate Water; PDW = Pacific Deep Water.

DOC concentrations in the ocean (Figure 1) range from a deep ocean low of ~35 μmolC/kg (in deep waters of the Pacific that were last exposed to the atmosphere and sunlight more than a millennium previously) to surface ocean highs of >80 μmolC/kg, with the highest concentrations commonly found in river-influenced coastal waters [1]. Biological processes establish the vertical gradient seen in Figure 1, with net autotrophic production in the sunlit surface layer and net heterotrophic consumption at depth, while physical conditions maintain the gradient (i.e., high vertical stability in the ocean water column largely precludes ready mixing of DOC-enriched surface waters to great depths).

Bulk DOC in the ocean is operationally resolved into at least three fractions, each qualitatively characterized by its biological lability [8]. All ocean depths contain (1) the very old, biologically refractory DOC (RDOC concentrations <45 μM and bulk radiocarbon ages of >6000 years) [14]. The RDOC distribution is controlled by the global deep overturning circulation and is thus relatively homogeneous in the deep ocean (Figure 1, note blues and pinks). Built upon the refractory DOC, at intermediate (to 1000 m) and upper layer depths, is (2) material of intermediate (or semi-) lability (SLDOC; lifetime of months to years; note greens, yellows, and reds in Figure 1). It is this material that has recently (years) accumulated in the surface ocean and that is then mixed downward into the ocean interior, thereby reducing the vertical concentration gradient and contributing to carbon export (i.e., the biological carbon pump; note great depth of green colors in the far left (near Iceland in the North Atlantic) of Figure 1). Concentrations of this fraction are commonly 10–30 μmolC/kg in the stratified upper ocean, and near zero in the deep ocean, indicating that it is susceptible to removal over decades. The most biologically labile fraction of DOC (3), with lifetimes of days to months and concentrations of just a few to 10′s of μmolC/kg, is largely limited to the sunlit layer of the ocean, where it is produced by autotrophs daily [2]. Referred to as labile DOC (LDOC), this material supports microbial heterotrophic processes in the surface ocean. Of the three fractions, it shows the greatest seasonality, with high net production rates during phytoplankton blooms and lower rates during the autumn and winter convective overturns of the upper ocean.

## 3. Global Distribution

There are two important perspectives taken when considering the distribution of DOC in the global ocean [1]. The first is in the wind driven upper layers; the wind forcing of the water column is at its strongest up to ~200 m depth, but it is still significant to depths up of 1000 m in some locations. The second is at greater depths (the deep ocean). The surface ocean is where most of the newly produced DOC that escapes fast microbial consumption (i.e., LDOC) accumulates (i.e., SLDOC). This material can then be delivered to great ocean depths as it is carried downward with the ocean’s overturning water masses; these waters ventilate the deep ocean over decadal to millennial time scales, carrying DOC with them (see section below).

**Figure 2:**
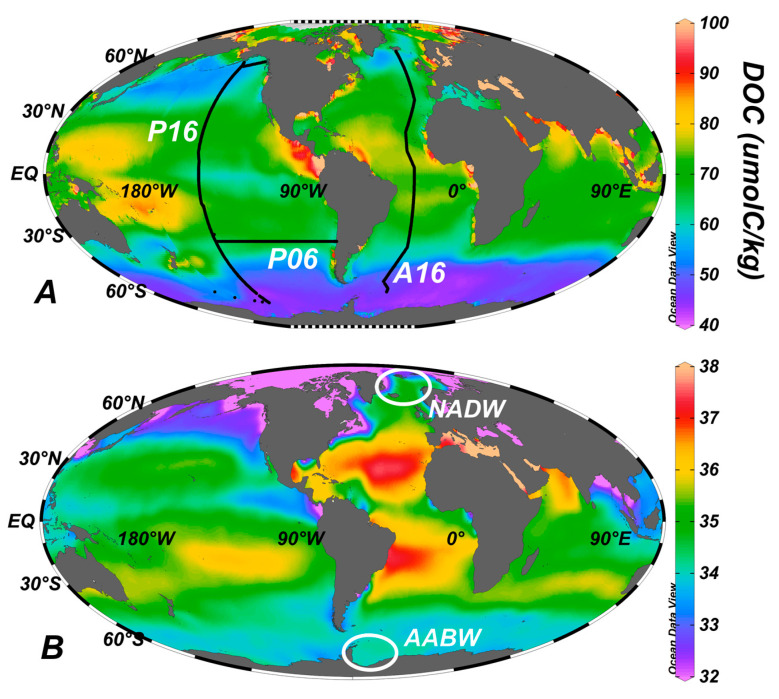
Modeled surface ocean distribution of (**A**) DOC (μmolC/kg) [15] and (**B**) gridded salinity (unitless) in August [16]. Ocean sections A16, P16, and P06 are also shown, the data for which are shown in Figure 1 and Figure 3. Figures were created using Ocean Data View [17]. White ellipses indicate formation locations of North Atlantic Deep Water (NADW) and Antarctic Bottom Water (AABW).

The distribution of DOC in the upper ocean is given in Figure 2. The highest concentrations are at low to mid-latitudes, while the lowest concentrations are common at higher latitudes, such as in the Southern Ocean (the Arctic Ocean is a strong exception to this rule, as it receives high loads of DOC created on land and that are delivered via the large Arctic rivers). Surface ocean waters at low to mid-latitudes are resistant to deep vertical mixing because the surface layer density is low due to solar warming, while the deeper underlying waters have high density due to very low temperatures (<3 °C, having originated in polar domains). Consequently, materials (e.g., DOC, heat, salt, planktonic organisms) present in those surface waters tend to remain there; they are not easily moved downward by vertical mixing. At higher latitudes, vertical stability is weaker (water density is high throughout the water column), so mixing is deeper, and the DOC produced at the surface can see its concentration reduced by that mixing.

## 4. Zones of Net DOC Production

**Figure 3:**
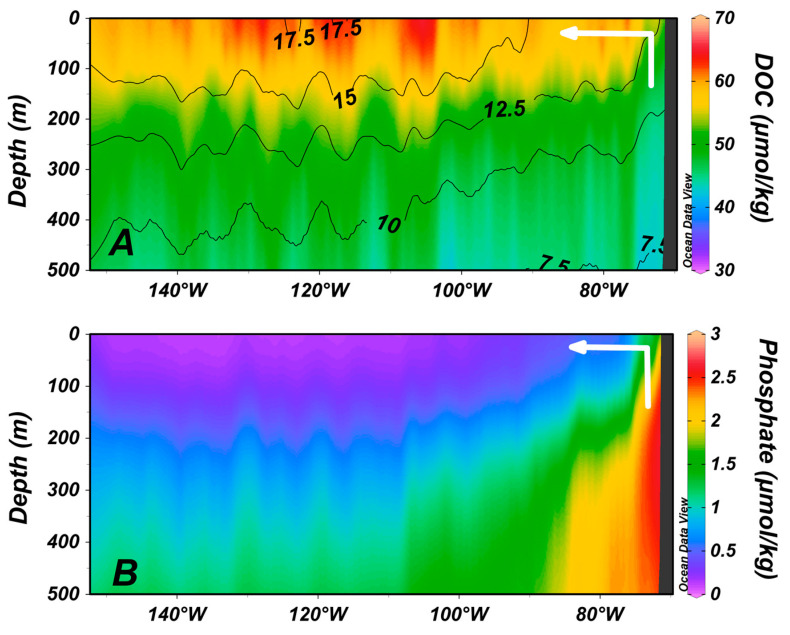
Vertical sections of (**A**) DOC (μmolC/kg) overlain by temperature contours (°C) and (**B**) phosphate (μmolP/kg) along ocean section P06 (see Figure 2 for location). White arrows schematically indicate upwelling along the coast of Chile.

It is important to note that concentrations of DOC in the surface ocean are not indicators of net DOC production rates in those locations. Instead, the rates of production are typically low in the most stratified waters (where DOC is elevated) because of the generally low nutrient concentrations there (i.e., oligotrophic conditions). It is largely the high vertical stratification existing in those nutrient impoverished waters that allows DOC to accumulate to elevated concentrations. The ocean systems producing DOC at the highest rates are typically found where the net productivity by autotrophs is high (i.e., eutrophic upwelling ocean systems) [18]. However, concentrations of DOC in upwelling systems are not typically high because the upwelled waters start with low initial DOC concentrations. However, the change in concentrations because of the upwelling can be large. In Figure 3, we see the vertical distributions of DOC (Figure 3A) and the nutrient phosphate (Figure 3B) off the coast of Chile in the South Pacific. Upwelling is evident by the uplift of high nutrient waters at the coast (note arrow in figure). Phosphate concentrations decrease upon reaching the euphotic zone due to net community production (NCP) in the system. One of the products of marine NCP is DOC; it increases in concentration (in the vertical) at the coast by ~10–15 μmolC/kg (from ~200 m to the surface). This concentration increase, as a product of NCP, is much larger than would be anticipated in the oligotrophic subtropical gyres. Net DOC production, as a function of NCP, is predictable [18,19,20].

## 5. Export of Surface Accumulated DOC to Depth with the Ocean’s Overturning Circulation

DOC accumulated in the upper ocean is susceptible to export down into the ocean interior by overturning circulation [1,21,22,23]. Such export occurs if the waters enriched in DOC reach the ocean zones that are seasonally made dense enough (through cooling or salinification) to be overturned, thus contributing to surface DOC export to the intermediate/deep ocean. To highlight the process, two ocean regions that are important for deep water formation are shown in Figure 2B. NADW forms north of Iceland, while AABW forms in the Weddell Sea (among other locations around Antarctica). In Figure 1 (far left in plot), we see that DOC-enriched waters are present (green color) throughout the water column; this distribution indicates that DOC was mixed downward during overturn associated with NADW formation, thus contributing to carbon export. NADW formation, then, is an important process for the export of DOC to great ocean depths. We do not see similar enrichments at depth in the Southern Ocean (center of plot in Figure 1) [24] despite the source of AABW being nearby. The difference between these two systems is that the Polar Frontal Zone of the Southern Ocean prevents DOC-enriched lower latitude surface waters from reaching the areas of bottom water formation. Evidence for the strong boundary created by the frontal system is the gradient in salinity (Figure 2B) from the subtropical gyres (green through red colors) to the Southern Ocean (blue colors). In contrast, the North Atlantic does not hold such a transport boundary; high-salinity and high-DOC lower latitude waters (such as from the subtropical gyre of the North Atlantic) are transported to the deep water formation region, thus favoring DOC export there [25]. There are several other deep and intermediate water formation sites in the ocean [26]; the role of each in DOC export similarly depends on the amount of DOC present in the surface waters at the initiation of the overturn.

Once the DOC-enriched deep waters (e.g., NADW) have formed, the excess DOC is slowly removed with time [20]. The longer the time since export, the less observable a decrease in concentration, though composition does show modest changes [27,28,29]. Whether the removal processes are biotic or abiotic remains to be demonstrated, as does the function of gels in deep DOC removal. Other papers consider DOC export by mechanisms such as convective overturn and release from sinking biogenic particles [23,30].

## 6. Zones of Deep Ocean DOC Enrichment Due to Sinking Biogenic Particles

In the section above, enhanced DOC concentrations in the deep ocean, such as in the far North Atlantic, were described as being due to introduction with the overturn of the water column. Biogenic organic particles sinking from the surface ocean likewise will introduce DOC to great depths [31,32,33,34]. Evidence for input by this mechanism is seen in Figure 1 (note dashed vertical arrows in the deep Pacific). The arrows are placed over small DOC enrichments (relative to surrounding waters) aligned vertically in the water column. As the particles sink, various biological and abiotic processes lead to their disaggregation and solubilization, with some of that material appearing as DOC [35]. This newly added DOC apparently has a limited lifetime (months) [34] because it serves as a substrate to the deep heterotrophic microbe populations [36]. We expect to find such enrichments spatially distributed throughout the global ocean and that are especially present where the export of large dense particles occurs, as these particles are more likely to reach the greatest depths of the ocean quickly. More slowly sinking particles typically do not reach great depths because they are intercepted by consumers or are disaggregated in the upper layers; hence, they will add DOC to those upper layers but to not the deep layers.

## 7. Composition of Ocean Dissolved Organic Matter

Dissolved organic matter (DOM) in the ocean can be categorized into two fractions based on radiocarbon ages and molecular composition. The first fraction is freshly produced, phytoplankton-derived DOM that is largely composed of classical biomolecules of known building blocks, such as polysaccharides, proteins, and lipids [37]. This is the material that is initially released by autotrophs in the euphotic zone, leading to accumulation there, and it is the material released into the deeper water column by sinking particles. Given its recent production, its radiocarbon age is modern. This modern material, which holds the LDOC and SLDOC fractions (described in Section 2), constitutes perhaps 3% of the oceanic DOC pool, with most being in the upper ocean layers.

The balance of oceanic DOC (97%) has a much greater radiocarbon age (>4000 years) and is absent in the classical biochemical character of the modern fraction. Most of the dissolved organic compounds in this older fraction have low molecular mass (<1000 Da) [38], and the chemical diversity is analytically challenging to characterize; the molecular structure of only a minor fraction of all of the compounds present is known. Estimates on the number of different compounds in DOM are inexact, but more than 20,000 molecular formulae have been identified with ultrahigh-resolution mass spectrometry, with 30 or more isomers per formula [39]. There are likely millions of different compound structures in DOM, each presumably below pico-molar concentrations. More detailed considerations of DOM composition are available in [37,40,41,42].

## 8. Closing with Consideration of Marine Gels

Microgels may account for an important fraction of DOC, having essential roles in shunting DOC polymers into particulate organic carbon (POC) through spontaneous assembly as well as providing polymer gel-rich substrates and habitats for bacterial biodegradation and remineralization. Gels have been proposed to play a pivotal role in regulating ocean basin-scale biogeochemical dynamics [6]. Marine gels link biological production at the ocean’s surface and microbial degradative processes at the ocean’s interior, cloud properties, radiative balance, and global climate [6].

Microgels exhibit unique physicochemical characteristics, such as reversible volume phase transitions stimulated by diverse environmental forcing conditions, such as pH [3,43], temperature [44,45], DMS and DMSP concentrations [4], solvent composition, light [46], etc. Though we still do not know the quantitative role of marine gels in the ocean carbon cycle, environmental effects on DOC biopolymers might have serious consequences for DOC dynamics and bioavailability. However, there are many issues that we still need to consider, such as what are the kinetics of polymer assembly, in situ? How does pressure affect assembly? What are the microgel distributions in the water column in the different oceans? What is their variability in the coastal ocean versus the open ocean? What are the ages of the polymer gels? What are the compositions of the assembled polymers, and how does it vary with depth? Answering these questions and many more will allow us to accurately understand the mechanisms involved in the transformation and dynamics of DOC polymers, and most importantly allow us to predict the effects of climate/ocean change and acidification on DOC dynamics. Marine polymer dynamics viewed in the context of soft matter physics can provide clear benefits for developing accurate models of the response of biogeochemical cycles to environmental forcing.

## Data Availability

Data plotted in the Figure 1 and Figure 3 are available at the NOAA National Centers for Environmental Information [47]. Data sources for Figure 2 are given in the caption.
